# Meditation training and non-native language training both reduce older adults loneliness in the age-well randomized controlled trial

**DOI:** 10.1038/s41598-025-21058-7

**Published:** 2025-09-29

**Authors:** Flora Hähnel, Antoine Lutz, Gaël Chetelat, Julie Gonneaud, Philipp Kanske, Olga Klimecki

**Affiliations:** 1 Faculty of Psychology, Technische Universität Dresden, Dresden, Germany; 2 Inserm, Lyon, France; 3 Normandie Univ, UNICAEN, INSERM, NeuroPresage Team, GIP Cyceron, U1237 Caen, France; 4 Universität Der Bundeswehr, Hamburg, Germany

**Keywords:** Psychology, Human behaviour

## Abstract

Loneliness is a key predictor for mortality and a risk factor for dementia. Meditation training appears to be a promising intervention for loneliness in older adults, but long-term randomized controlled trials with an active control group are scarce. To fill this gap, this secondary analysis of the three-armed clinical randomized controlled Age-Well trial compares the impact of an 18-month meditation training to an 18-month non-native language training, and a passive control group on self-reports of loneliness and post-training reactions to experimentally induced social exclusion in 137 cognitively unimpaired community-dwelling older adults (age ≥ 65 years). Multilevel models revealed that both interventions reduced loneliness from pre- to post-intervention compared to the no-intervention group. No group differences were observed regarding self-reported emotional reactions to social exclusion. Taken together, our results suggest that sharing regular activities with the same group of persons for a long period reduces older adults’ loneliness.

**Supplementary Information:**

The online version contains supplementary material available at 10.1038/s41598-025-21058-7.

## Introduction

With increasing life expectancy, promoting healthy aging is an increasingly important topic in health care policy. Feeling lonely or socially excluded has detrimental effects on longevity and mental health, and can increase the risk for dementia^1–4^. In the last years, the impact of mindfulness-based interventions^5–7^ and other non-pharmacological interventions^8^ on loneliness in older adults have been shown to be effective. Still, sufficiently powered causal evidence on the impact of meditation and cognitive training on the reduction of older adults’ loneliness and on changes in reactions to social exclusion is missing. Thus, the present Age-Well intervention study^9^ tests the effects of long-term meditation or non-native language training on the secondary outcome measures of loneliness and social exclusion in a sample of community-dwelling older adults.

The Age-Well randomized controlled trial, embedded in the European project Medit-Ageing/Silver Santé Study, is a three-armed superiority trial that compares the effects of meditation training to an active control group (non-native language training, *i.e.,* English) and a no-intervention group. The primary outcome showed no significant change in grey matter volume and perfusion of anterior insula and anterior cingulate cortex^10^. Secondary outcomes were various self-report measures and a range of biological and behavioral measures. First longitudinal results show no significant effects of meditation training or non-native language training on cognition^11^ and selective improvements of meditation training in domains of psychological well-being^12^. Here, we study the impact of the different interventions in the Age-Well trial on loneliness and the response to social exclusion.

Loneliness, defined as the subjective experience of “an unpleasant or inadmissible lack of (quality of) certain relationships”^13^, affects a substantial part of older people. The proportion of older adults experiencing severe loneliness ranges from 2 to 9%, while 30% to 39% report feeling lonely occasionally, based on data from older populations in England, Ireland, Finland, and Australia^14–19^. Large sample studies indicate that loneliness is relatively low in the beginning of old age (aged 60 to 80), whereas it reaches its highest levels among the oldest old(80 years and older)^15,20,21^. Loneliness has detrimental effects on healthy aging, being associated with a higher risk of Alzheimer’s disease and dementia^3,4^, more depression and lower life satisfaction^16^, as well as increased mortality^2^.

Social exclusion or ostracism describes being ignored and excluded by individuals or groups without an explicit declaration that the ostracised person is unwanted^22^. A common paradigm for the experimental manipulation of ostracism is the Cyberball paradigm^23^. Cyberball is a virtual ball-tossing game that participants allegedly play with other players. To study the effects of ostracism, the frequency of how often the ball is tossed to the participants is manipulated^23^. Despite this minimal design, participants care about how often they get the ball – being ostracized by Cyberball deteriorates participants’ needs for self-esteem, belonging, control, and meaningful existence^24,25^. Currently, older adults are an underrepresented group in the research on social exclusion^26,27^. Yet, studying the impact of social exclusion in older adults is important, as several factors such as restricted mobility, less material and financial resources, health constraints, loss of social roles, and less intergenerational living make older adults vulnerable to social exclusion^28–31^. In fact, network sizes are consistently found to decrease with age^32^. However, older adults report ostracism experiences less frequently than younger adults^33^. Hawkley and colleagues^34^ found that older adults experienced less negative affect, more positive affect, and felt less frustrated in their needs than younger participants in reaction to Cyberball, although older and younger adults did not differ in the detection of ostracism. This finding might be explained by older adults’ “positivity bias”^35^.

To summarize, while feelings of loneliness increase as people age, the experience, or the impact of ostracism appears to decline with age.

## Mindfulness and loving kindness meditation

As stated earlier, feeling socially connected is crucial for healthy aging. Thus, effective interventions to reduce loneliness in older age are needed. One promising approach is mindfulness training^36^. Mindfulness is described as paying attention in a particular way: in the present moment, without judging, without reacting, and with an open heart^37^. Mindfulness meditation and loving kindness meditation, a practice that promotes feelings of kindness and benevolence towards oneself and others^38^, are found to increase feelings of connection^39–43^. A recent study focused on the variability of social connectedness over 11 weeks and found that both, mindfulness meditation and loving kindness meditation lead to more stable relationships^44^. Since the latter study^44^ did not compare the interventions to a passive or active control group, the findings need to be interpreted with caution.

Some findings also indicate a positive effect of meditation on loneliness. Lindsay and colleagues^5^ implemented a three-arm smartphone-based intervention on young to middle-aged adults that either trained solely monitoring, or monitoring and accepting, present thoughts and feelings, or an active coping control group (*i.e.*, free reflection, analytic thinking, and problem solving). Only the monitoring and acceptance group reported a decline in loneliness as well as an increase in social contacts^5^. Older adults remain an underrepresented group in the research of meditation. Only two studies that examined the effects of meditation training on loneliness in older adults were found. A small randomized controlled trial found that Mindfulness-Based Stress Reduction training reduced loneliness in older adults, compared to a waitlist control group^6^. Pandya^7^ compared older adults that underwent a two-year meditation training with a no-intervention group and found that meditation training reduced loneliness and increased well-being, life satisfaction, and contentment. Home practice most strongly predicted the outcomes^7^. Unfortunately, both studies on older adults lack an active control group.

Saini and colleagues^45^ conducted a scoping review on the effect of various meditation practices on loneliness of participants aged over 18 years. Although 11 of 13 studies on the effects of various meditation techniques on loneliness reported positive results, the authors criticize the small sample sizes and the heterogeneity of measurements and definitions of loneliness^45^. The review of Saini and colleagues^45^ included studies with diverse study designs. Five of the included studies used an active control group (e.g^43^.,), three studies compared the intervention to a passive control group (e.g^7^.,), three studies used a waitlist control group (e.g^6^.,) and two studies did not compare the intervention effects to a control group^46,47^. Similarly, an umbrella review identified meditation/mindfulness as one of three successful interventions in reducing loneliness, but stated that the evidence is low or very low^48^. The aforementioned systematic review^8^ analyzed 60 studies on various non-pharmacological interventions on older adults’ loneliness, thereof only one on the effects of meditation training^7^. The authors included studies that used either an active control group, usual care, or a no-treatment control group in the systematic review^8^. They found that the six studies they grouped as “psychological interventions”, including counselling-based psychological interventions, the study on meditation training^7^ and a study on yoga^49^, had a large effect on reducing loneliness (Hedge’s = − 2.33) and were more effective than the other interventions. In a further analysis, they found the counselling-based psychological interventions to be more effective than the two studies on yoga and meditation. Thus, robust data comparing the effects of meditation training on loneliness of older adults to an active and a passive control group are needed.

By observing and accepting the present moment as it is, mindfulness is thought to prevent rumination. This could also apply to social exclusion experiences. Indeed, several studies found that trait mindfulness and mindfulness training were associated with better mood and less need threat after Cyberball^50–52^. In addition, as mentioned above, several studies on meditation have found that meditation training increases feelings of connection^39–43^, potentially reducing the harmful experience of social exclusion. More generally, mindfulness- and compassion-based interventions can improve emotionregulation^53^, which might contribute to reducing negative affect and distress and increasing positive affect^54^. Whether this applies to experiences of ostracism in older adults is an open question. No study was found that examined the effects of a long-term meditation training, or effects of meditation on Cyberball on a sample of older adults. No study was found that analyzed the impact of loving-kindness meditation on ostracism experiences, although it is plausible that the feelings of kindness and connectedness that are cultivated through loving-kindness meditation can buffer the negative effects of being socially excluded.

To summarize, research on the ostracism experience of older adults is needed, as older adults are an underrepresented group in the study of ostracism. Research on the effects of meditation training on older adults’ reaction to ostracism is missing to date. Furthermore, studies on the effects of meditation training on loneliness often use a small sample size, short-term interventions without a follow-up measure, and passive control groups^45^. Randomized controlled trials with large sample sizes, an active control group, and a long-term follow-up are needed to study the effects of meditation on loneliness more thoroughly.

## Research objective

To address these shortages, this group-based randomized controlled study tested the changes in loneliness and social exclusion as secondary outcomes of the Age-Well intervention study. In contrast to most existing studies, the current study uses a large sample size and compares meditation training not only to a no-intervention group, but also to an active control group, that is, non-native language training. Using an active control group allows to attribute findings more directly to the meditation training and reduces the risk of measuring only the impact of non-specific elements, such as regular social contact, cognitive mental training, or treatment expectancies^9^. Furthermore, the current study exceeds other studies by its long intervention period of 18 months and its three included measurement points, namely pre-intervention, mid-intervention, and post-intervention. The second measurement time mid-intervention allows to differentially study the effects of two meditation techniques on loneliness: Mindfulness meditation, which is taught in the first nine months, and loving kindness and compassion meditation, practiced in the following nine months. Based on previous findings on the potentially buffering effect of mindfulness, we hypothesized that meditation training will significantly alleviate the negative effects of social exclusion. More precisely, we expected that in response to ostracism, older participants in the meditation group will report higher levels of positive affect, lower levels of negative affect, and lower levels of distress, as compared to a non-native language training and a no-intervention group. Furthermore, we hypothesized that meditation training and non-native language training will both significantly decrease loneliness compared to a no-intervention group due to regular social contact. Indeed, a review described that non-native language training in later life could promote social interaction and integration^55^. Based on previous findings on loneliness and social connectedness, however, we hypothesized that meditation training will reduce loneliness significantly more than non-native language training. With planned explorative post hoc analyses, we also investigated whether sex, age, and practice time influence the results.

## Methods

### Sample and procedure

The current study is based on the sample of the Age-Well randomized controlled study that evaluated the impact of an 18-month meditation or non-native language training on emotional regulation and cognitive control of 137 cognitively unimpaired older adults^9^. The sample size calculation was based on the primary outcome, which compared the meditation versus passive control arms on the mean change in (1) volume and (2) perfusion of the anterior cingulate cortex and insula from the baseline pre-intervention visit to the end of the 18-month intervention^10^. Based on a meta-analysis of meditation effects on neuroimaging markers^53^, we aimed to demonstrate an effect size of 0.75 for each of the four comparisons (volume and perfusion for anterior cingulate cortex and insula each), with 80% power and a two-sided type I error of 1.25% (Bonferroni correction for test multiplicity). Thus, 42 participants per arm (at least 126 participants in total) needed to be included. In three recruitment waves, participants were recruited from the general population in Caen, France, and invited to a screening visit (V0). Participants who met inclusion criteria (e.g., age ≥ 65 years, living at home, native French speaker, no present or past regular or intensive practice of meditation) were invited to a baseline visit pre-intervention (V1). Exclusion criteria were, among others, major neurological or mental disorder, history of cerebral disease, and current medication that could interfere with cognitive functioning (see Poisnel et al.^9^ for a full list of exclusion criteria). A total of 83 women (60.58%) and 54 men (39.42%) were included, with a mean age of 68.4 years (*SD* = 4.0, *Mdn* = 67, range 63–83) at V0 and 13.2 years of education (*SD* = 3.1, *Mdn* = 14, range 7–22). The most common level of occupation participants have had in the past was “professionals” (30.66%), followed by “technicians and associate professionals” (21.90%), and “clerical support workers” (17.52%). All participants were retired at V0, for 8.6 years on average (*SD* = 5.3, *Mdn* = 8, range 1–25). Participants were enrolled by the study team between November 24, 2016, and March 5, 2018, in France. Participants were randomized to each of the study arms after their baseline assessment with the ratio 1:1:1. The randomization list with permuted blocks of varying size (6 and 9) was generated centrally by a biostatistician at the EUCLID clinical trials platform. All study employees, except for teachers, trial-independent statisticians, and data monitoring infrastructure staff, were blinded to treatment allocation. Nine months after the intervention start, a mid-intervention visit (V2) was performed, and after another nine months, a post-intervention visit (V3) was realized. All participants gave informed written consent. The Age-Well randomized controlled trial conformed to the Declaration of Helsinki and was approved by the ethics committee (CPP Nord-Ouest III, Caen; EudraCT: 2016–002,441-36; IDRCB: 2016-A01767-44) and registered on ClinicalTrials.gov (NCT02977819) on the 25^th^ of November 2016. A more detailed description of the procedure can be found in the trial protocol paper by Poisnel and colleagues^9^.

### Intervention

Interventions were conducted in groups of 14 to 17 participants. Participants of the intervention groups were asked not to practice the activity of the other intervention group. Participants of the no-intervention group were asked not to change their habits and not to practice either meditation or non-native language training. Both interventions were matched in overall course length, class time, home activities, and the number and expertise of teachers. The programs were furthermore matched in administration, dosage, and duration. Researchers’ allegiance to the two interventions was actively balanced through equal communication on expected results. Participants with strong aversion or preference for one intervention were not invited to V1. The two interventions consisted of a two-hour weekly group sessions, daily home practice for at least 20 minutes via an application on a tablet, and one day of intensive practice for five hours.

The meditation training program was specially designed for the Silver Santé Study and is based on previous interventions with a focus on personal development and healthy aging (for details see Poisnel et al.^9^). The meditation training aims at helping participants to develop mindfulness, kindness, and compassion. The program was split into nine months of mindfulness training, followed by nine months of loving-kindness and compassion meditation training, similar to the Cognitive-Based Compassion Training from Emory University^56^. The rationale is that cultivating mindfulness and the capacity to voluntarily direct one´s attention are seen as the basis for then cultivating compassion towards oneself and others. Mindfulness can be helpful to calm down when compassion practices trigger difficult feelings or distress^56^. Teachers were expert meditation instructors. Every session included group meditation, sitting, or walking, as well as teaching and sharing. The non-native language learning program aimed atimproving participants’ understanding, speaking, and writing. The teachers’ level of expertise was matched to the expertise of the meditation instructors. The non-native language intervention was hypothesized to enhance cognitive control but not emotion regulation, while the meditation intervention was hypothesized to improve both, cognitive control and emotion regulation.

### Measures

Social exclusion was experimentally manipulated with the Cyberball paradigm^23^. In a laboratory session, participants were told that they would play an online game with other players who were connected via internet. They were asked to mentally visualize the entire experience, including the other players. Participants of all groups were equally ostracized through the three-minute game by receiving the ball in only two out of 30 throws. Two minutes before and after playing Cyberball, participants answered written questionnaires with single-item ad hoc measures of positive affect, negative affect, and distress (e. g., “Please indicate the intensity of your positive (negative) emotions”, “To what degree did you feel distressed during the game?”). Participants rated their answers on scales from “not at all” or “neutral” (coded as zero) to “very distressed”, “very negative”, or “very positive” (coded as ten). Due to the ethical responsibility to debrief participants about the manipulation, Cyberball was only administered once, at V3, to test intervention effects. Debriefing of the participants included the question how they experienced the Cyberball task. None of the participants revealed not believing the cover story.

Loneliness was measured with the Three-Item-Loneliness-Scale (α = 0.72)^57^. Participants filled in the questionnaire at home. The Three-Item-Loneliness-Scale measures loneliness with the items “How often do you feel that you lack companionship?”, “How often do you feel left out?”, and “How often do you feel isolated from others?”. All questions can be answered with one (“hardly ever”), two (“sometimes”), or three (“often”). Values were summed up to a total score of loneliness, ranging from three to nine. Loneliness was measured at each time point (V1, V2, and V3).

Treatment credibility and expectancy of the interventions were measured at V1 with the Credibility and Expectancy Questionnaire (6 items, α = 0.84 − 0.85)^58^. A variable for practice time in minutes and a dichotomous variable for responders versus non-responders were created. For details on these measures, see eCompliance in the Supplements.

### Data analyses

Linear mixed models with maximum likelihood estimation were conducted to analyse the effects of the intervention group and measurement time (V1, V2, V3 for loneliness and pre and post Cyberball at V3 for social exclusion) on the outcomes loneliness and social exclusion using the “lme4” and “lmerTest” packages of R 4.0.0^59–61^. Model assumptions (multicollinearity and homoscedasticity of residuals) were given. Models were specified as random intercept models with fixed slopes with the time-variant predictor “measurement time” at level 1, and the time-invariant predictor “group” at level 2. The parameter of interest was the cross-level interaction between intervention group and measurement time. Following model estimation and diagnostics, the fixed effects of the model were inspected with a Type II analysis of variance (ANOVA) breakdown, using F-tests with Satterthwaite’s correction to the degrees of freedom. Post-hoc pairwise contrasts were conducted when significant effects emerged to see which contrasts drove the significant effect. For exploratory analyses, a covariate for the three recruitment waves, a dummy-coded covariate for sex (0 = male; 1 = female), and mean-centered covariates for age and years of education were included to assess the main effects of these covariates. Marginal R-squared values (*R*^*2*^_*m*_) were calculated to see how much variance was explained by the models. Furthermore, correlation analyses between practice time and change in loneliness from V1 to V3, as well as between practice time and the difference in affect and distress from pre to post Cyberball questionnaires were conducted for both intervention groups. The no-intervention group did not receive training and was consequently excluded from analyses with practice time. Sensitivity analyses were conducted excluding participants with a session attendance of less than 20% and excluding non-responders. Influential cases were detected using Cook’s distance, with as threshold the 50% quantile of the distribution *F*(*P*, *N* − *P*), with P the number of fixed effect parameters in the model and N the total sample size. Fixed effects were considered significant at a level of α = 0.05 and 95% confidence intervals were estimated.

Mann–Whitney U tests with a significance level of α = 0.05 were conducted to analyse differences between intervention groups in class attendance, practice time, credibility, and expectancy of the intervention. Fisher’s exact test was conducted to analyse intervention group differences in the number of responders.

## Results

### Sample characteristics

**Fig. 1:**
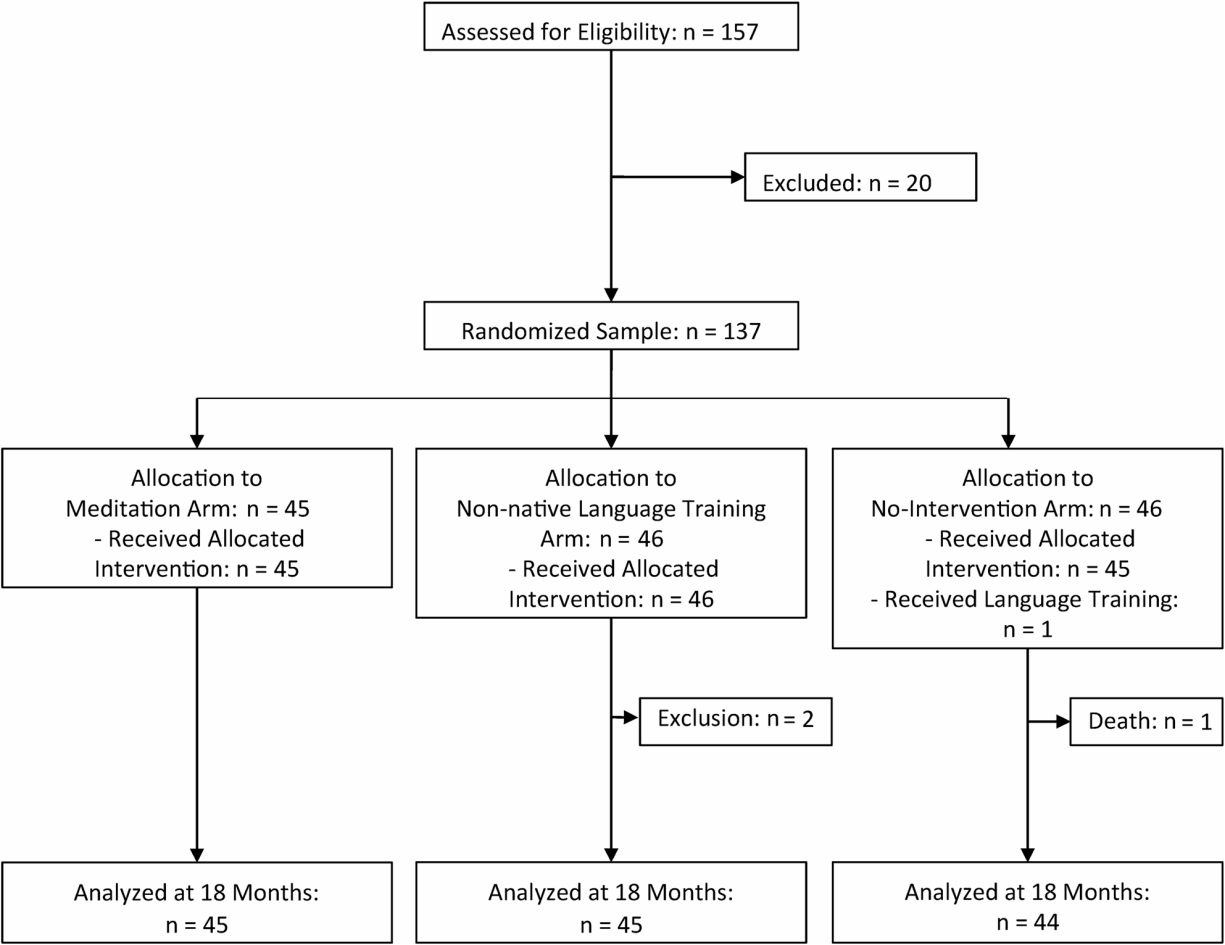
Flowchart of participants. One participant did not follow his allocated treatment (no-intervention) and was analyzed as a participant in the intervention he actually received (non-native language training). One participant died in the course of the study. Her baseline values were included in the analyses but could not be analyzed at 18 months.

Recruitment took place in three recruitment waves from November 24^th^, 2016, to March 05^th^, 2018, and the post-intervention measures (V3) ended in early 2020. Of 157 older adults screened for eligibility, 137 participants were randomized to the allocation arms (45 participants in the meditation group, 46 in the non-native language training group, and 46 in the no-intervention group). Two participants were excluded because they did not meet eligibility criteria. One participant died in the course of the study. Her baseline values were included in the analyses. One participant did not follow his allocated treatment (no-intervention) and was analyzed as a participant in the intervention he actually received (non-native language training). Of the remaining 135 participants, all contributed data on the outcome loneliness, and 133 provided data on the effects of social exclusion. Due to logistic issues, one participant only answered the questions on affect and distress before the Cyberball game. This participant’s missing values are therefore expected to be missing at random. Figure 1 depicts the flow of participants. No influential cases were found using Cook’s distance. Analyses on intervention compliance can be found in the Supplement (eCompliance).

### Effect on loneliness and social exclusion

**Fig. 2:**
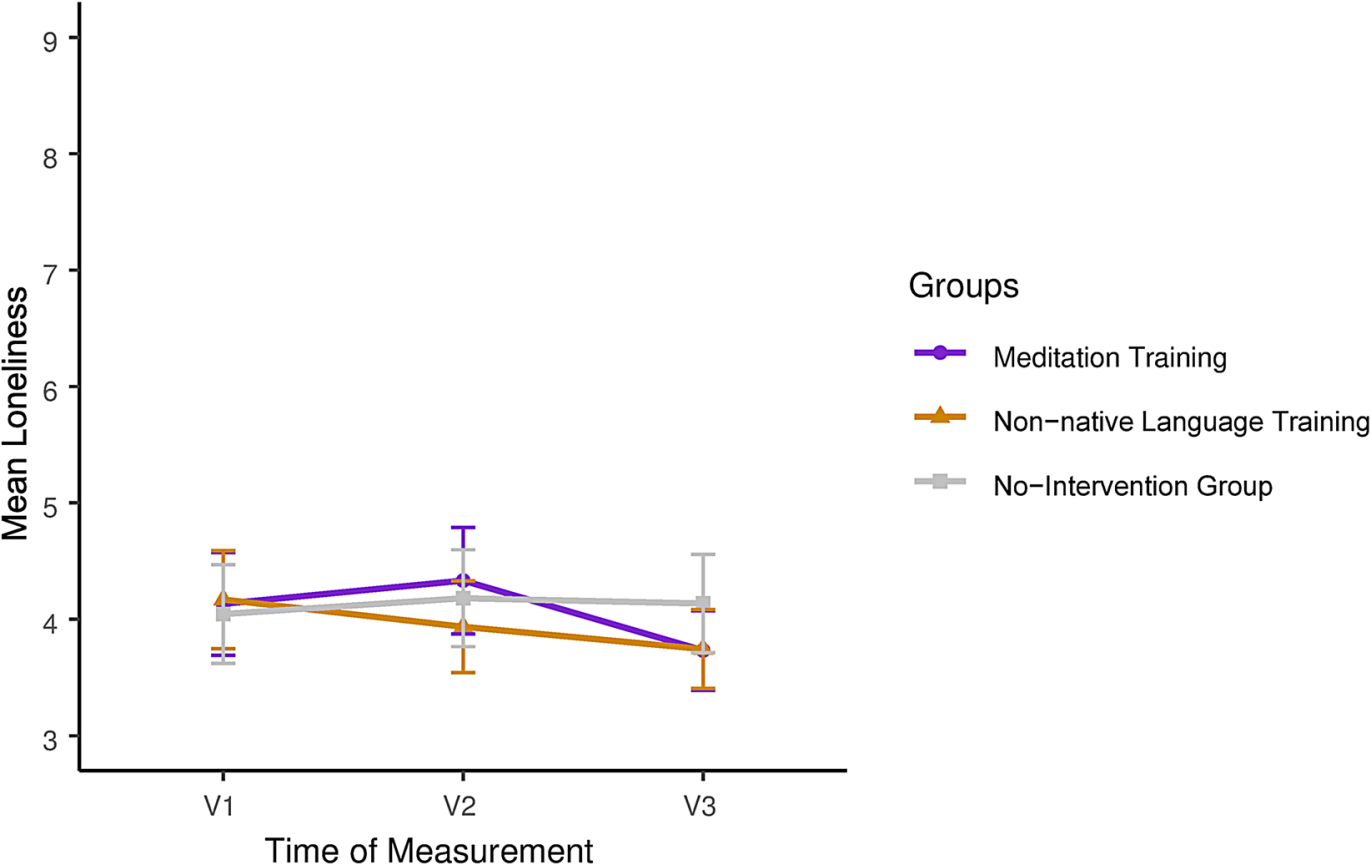
Longitudinal trajectories of loneliness by trial arm.

Descriptive values of loneliness are presented in Table 1, while group-specific trajectories are displayed in Fig. 2. The random intercept model of the outcome loneliness revealed a significant interaction effect (*F*(4, 268.47) = 3.26, *p* = 0.012) between both intervention groups and time at V3 (meditation group: ß = − 0.50, *SE* = 0.22, 95% CI [− 0.92, − 0.07], *t*[268.86] = − 2.3, *p* = 0.022; non-native language group: ß = − 0.52, *SE* = 0.22, 95% CI [− 0.95, − 0.09], *t*[268.86] = − 2.4, *p* = 0.017), relative to the no-intervention group. Explained variance was low (*R*^*2*^_*m*_ = 0.02). Post-hoc pairwise contrasts showed significant decreases in loneliness between V1 and V3 for both intervention groups (meditation group: *estimate* = 0.4, *SE* = 0.15, CI [0.10, 0.70], *t*[268.2] = 2.63, *p* = 0.009; non-native language training group: *estimate* = 0.42, *SE* = 0.15, 95% CI [0.12, 0.72], *t*[268.2] = 2.8, *p* = 0.006) as well as between V2 and V3 for the meditation group (*estimate* = 0.6, *SE* = 0.15, 95% CI [0.30, 0.90], *t*[268] = 3.94, *p* < 0.001). Loneliness in the no-intervention group did not decrease significantly between V1 and V2 (*estimate* = − 0.14, *SE* = 0.15, CI [− 0.44, 0.17], *t*[269.5] = − 0.89, *p* = 0.377), nor between V2 and V3 (*estimate* = 0.05, *SE* = 0.15, CI [− 0.26, 0.35], *t*[268.2] = 0.30, *p* = 0.768).

**Table 1:** Descriptive statistics for the outcome loneliness.

	Pre-intervention (V1)		Mid-intervention (V2)		Post-intervention (V3)	
	*M*	*SD*	*M*	*SD*	*M*	*SD*
Meditation arm	4.1	1.5	4.3	1.5	3.7	1.1
Non-native language training arm	4.2	1.5	3.9	1.4	3.8	1.2
No-intervention arm	4.0	1.4	4.2	1.4	4.1	1.4

Pertaining to the reaction to ostracism, linear mixed models revealed no significant effects of study arm (see Table 2 and the Supplement eExploratory Analyses).

**Table 2:** M*eans*, standard deviations, and one-way analyses of variance for the outcomes positive affect, negative affect, and distress.

Outcome		Pre Cyberball		Post Cyberball		Group		Time		Group x time interaction		
		*M*	*SD*	*M*	*SD*	*F*	*p*	*F*	*p*	*F*	*p*	*R*^*2*^_*m*_
Positive affect	Meditation arm	6.3	2.7	5.4	3.2	*F*(2, 133.19) = 1.35	.262	*F*(1, 131.64) = 1.41	.237	*F*(2, 132.63) = 2.07	.131	.02
	Non-native language training arm	6.5	2.7	6.4	2.6							
	No-intervention arm	5.5	2.6	5.7	2.9							
Negative affect	Meditation arm	2.8	2.4	2.9	2.9	*F*(2, 131.95) = 0.43	.650	*F(1, 131.51) = 1.14*	.288	*F*(2, 131.51) = 0.55	.578	.01
	Non-native language training arm	3.1	2.8	2.7	2.7							
	No-intervention arm	2.8	2.8	2.3	2.6							
Distress	Meditation arm	2.3	2.7	1.8	2.4	*F*(2, 131.97) = 1.03	.359	*F*(1, 131.56) = 5.10	.026*	*F*(2, 131.56) = 0.36	.700	.02
	Non-native language training arm	2.6	3.0	1.6	2.5							
	No-intervention arm	1.6	2.7	1.3	2.0							

Further analyses and results of exploratory analyses can be found in the Supplement (eExploratory Analyses). Sensitivity analyses excluding participants with < 20% session attendance (n = 1) or non-responders (n = 9) did not substantively change results.

## Discussion

The present study evaluated the impact of an 18-month meditation training, an 18-month non-native language training, and a passive control group on older adults’ loneliness and reaction to social exclusion. With a three-armed randomized controlled design and a long intervention period, this study provides important causal findings on the effects of meditation and non-native language training on the reduction of loneliness – a key risk factor for mortality – in older adults. These findings enable policymakers in the healthcare domain to implement preventive strategies that can promote well-being and longevity in older adults.

We show that both interventions significantly reduced loneliness from pre- to post- intervention compared to the no-intervention group. In the domain of ostracism, this first study on the effects of meditation training on older adults’ reaction to ostracism through the Cyberball paradigm showed no significant differences between trial arms in older adults’ response to being ostracized on positive affect, negative affect, and distress.

Other studies have also found that meditation training is effective in reducing older adults’ loneliness^6,7^. With our three-armed study design, we were able to place these results into perspective by finding that not only the meditation training but also the non-native language training reduced participants’ loneliness. This finding indicates that it is not the meditation training itself that reduces loneliness, but probably aspects that both interventions have in common, such as the regular social contact with peers and teachers with the opportunity to create new and strong social relationships. From this, one might conclude that when it comes to reducing loneliness, the training content is not as important as meeting other people regularly. It has been previously shown that the quantity of social contacts is negatively associated with loneliness in all age groups^20^, and social network growth is a protective factor against loneliness in old age^62^. Our results also extend the broaden-and-build-theory as well as previous findings that loving kindness and compassion meditation increase received social support^63^, by showing that the training of loving kindness and compassion meditation in the last nine months specifically decreased loneliness.

It is important to note that the present study’s sample experienced very low levels of loneliness at all measurement times, as can be seen in Table 1. This indicates that the current sample is socially well integrated. Moreover, the low levels of loneliness are in line with the findings of several studies that have found high levels of loneliness predominantly among the oldest old, that is, 80 years and older^15,20,21^. Although the current sample’s age ranged from 63 to 83 years, only one participant was older than 80 years. Pinquart and Sörensen’s meta-analysis^21^ found no association between age and loneliness in the age group from 60 to 80 years.

All groups reported rather high positive affect, low negative affect, and low distress to the ostracism experience through the Cyberball task. Trial arm did not have a significant influence on the reaction to the ostracism experience. This might be because the “other players” were strangers to participants. Since the differentiation between intimate and unfamiliar relationships gets increasingly important in old age^64^, social exclusions by friends or family members might have affected participants’ emotional responses more strongly. The positivity bias in older age might also explain participants’ equanimous reaction to the experience of ostracism^35^.

Finally, it is important to consider the very special Age-Well study sample. Despite the long study period of nearly two years covered here and the extensive behavioral measurements at three measurement times with additional neuroimaging, sleep, and biological measurements at two measurement times, the participants who dropped out were due to death (1 person) or exclusion criteria (2 persons). Compared with often reported drop-out rates of 30–70% in longitudinal studies^65^, this indicates a highly motivated and altruistic sample.

In contrast to most research on meditation training, the current study uses a three-armed clinical randomized controlled design with a large sample that allows reliable insight into the effects of meditation training. Although rarely used, comparing meditation training to both, an active control group and a no-intervention group provides more robust data on the effects of meditation training. The long intervention period of 18 months also stands out from most other studies with shorter intervention periods^5,6^. Moreover, the present study tested a sample of older adults which, to our knowledge, has not yet been the subject of research when exploring meditation effects on response to the Cyberball paradigm. Similarly, very few studies analyzed meditation effects on loneliness with a sample of older adults^6,7^. While van Dam and colleagues^66^ warn of the risk of bias through researchers’ allegiance and complain about insufficient descriptions of meditation interventions, the current study activelybalanced researchers’ allegiance and described not only the meditation intervention but also the non-native language training in detail^9^. In line with recommendations by Kreplin and colleagues^67^, the primary endpoint assessments were blinded, and expectations of participants were measured. By splitting the 18-month meditation intervention in 9 months of mindfulness meditation training and 9 months of loving-kindness and compassion training and collecting data in between, the specific and the common effects of both meditation techniques can be analyzed. Indeed, mindfulness meditation and loving-kindness and compassion meditation are hypothesized to reinforce each other: While mindfulness meditation down-regulates habits and negative thinking patterns, loving-kindness and compassion meditation upregulates positive emotions^68^. Importantly, the intervention teachers were not co-authors of the study. The very low dropout rate shows that the sample was highly motivated to take part in the study, even though it was very time-consuming.

### Limitations

The unusually low dropout rate, despite the long intervention period, indicates a highly motivated sample, which diminishes the generalization to other populations. However, the recruitment from the general population and the randomized allocation to trial arms suggest generalizability to older adults who are willing to take a course in meditation or non-native language training for a rather long time. The Three-Item-Loneliness Scale^57^ is an economical measure of loneliness that shows satisfactory reliability and validity. Nevertheless, objective measures of loneliness, such as measuring daily contacts, could have helped to draw a more complete picture of the effects of meditation training on loneliness.

## Conclusion and outlook

In times of demographic changes, policymakers in the health-care sector need to have causal evidence on which interventions can reduce key risk factors for mortality in older age. The Age-Well randomized controlled trial shows that compared to a passive control group, loneliness, which is a key risk factor for mortality, was decreased by two non-pharmacological interventions (meditation training and non-native language training) from pre-intervention to post-intervention 18 months later. At the same time, no differences between study arms were found in response to being ostracized by strangers. We conclude that regular group-based meetings are important to reduce older adults’ loneliness and consequently to foster public health. In the meditation group, it was particularly the loving-kindness and compassion component that decreased loneliness.

## Supplementary Information

Supplementary Information 1.

Supplementary Information 2.

## Data Availability

The data underlying this report are available on request following a formal data sharing agreement and approval by the consortium and executive committee (https://silversantestudy.eu/2020/09/25/data-sharing/). The material can be mobilized, under the conditions and modalities defined in the Medit-Ageing Charter, by any research team belonging to an Academic Institution for carrying out a scientific research project relating to the scientific theme of mental health and well-being in older people. The material may also be mobilized by non-academic third parties, under conditions, in particular financial, which will be established by separate agreement between Inserm and the said third party. Data sharing policies described in the Medit-Ageing Charter are in compliance with our ethics approval and guidelines from our funding body. When requesting data from the present study, please contact the corresponding author (OK).
