# Supplementary material for: Meditation training and non-native language training both reduce older adults loneliness in the age-well randomized controlled trial
Source: Sci Rep. 2025 Sep 29;15:33332. doi: 10.1038/s41598-025-21058-7 (PMC12480588; doi:10.1038/s41598-025-21058-7)
Supplement: Supplementary file 1 — Supplementary Information 1. [file 41598_2025_21058_MOESM1_ESM.docx]

**Supplemental Material**

1. eCompliance (p. 2)
2. eExploratory Analyses (p. 3)

**eCompliance**

**Measures**

Treatment credibility and expectancy of the interventions were measured at V1 with the Credibility and Expectancy Questionnaire (6 items, α = 0.84 - 0.85; Devilly & Borkovec, 2000). This questionnaire assesses credibility of the intervention with three items asking what participants think about the intervention (e.g., “So far, how helpful do you think this intervention will be to you in aging better?”). Participants’ expectancy of the intervention is measured with one item (“How much of an impact do you think it will have on your well-being after the 18-month intervention?”). The questionnaire also assesses perceived credibility of the intervention (“So far, how useful do you feel this procedure will be for you to age well?”) and perceived expectancy of the intervention (“How much of an impact do you feel it will have on your well-being after the 18-month intervention?”). Answer options ranged from 0 % to 100 %, except for the scale assessing perceived credibility that ranged from 1 to 9. Practice time is specified in total minutes of class attendance (60 minutes per session) combined with reported formal home practice. Intervention performance and changes in participants were assessed by teachers using a global meditation composite or English test scores from V1 to V3. These scores were used to create a dichotomous variable for responders versus non-responders.

**Results**

The reported class attendance ranges from 26 to 70 in the meditation group and from 7 to 68 in the non-native language group, with no significant differences between intervention groups (meditation group: M = 60.6, SD = 8.2; non-native language group: M = 58.0, SD = 12.3; p = .252). The meditation group reported more practice time than the non-native language group (meditation group: M = 12,821, SD = 6113.6; non-native language group: M = 8,783.7, SD = 4045.0; p < .001). Intervention groups did not significantly differ in number of responders (meditation group: n = 38; non-native language group: n = 43; p = 0.157). Both intervention groups reported rather low credibility of the intervention (meditation group: M = 22.8, SD = 3.2; non-native language group: M = 23.5, SD = 3.5), but high perceived credibility of the intervention (meditation group: M = 7.6, SD = 1.1; non-native language group: M = 7.6, SD = 1.5), with no significant between-group differences (credibility: p = .260; perceived credibility: p = .815). Participants expected a positive impact of the interventions on well-being, as reflected in rather high values in expectancy (meditation group: M = 75.1, SD = 14.9; non-native language group: M = 69.3, SD = 17.5) and perceived expectancy (meditation group: M = 74.9, SD = 16.6; non-native language group: M = 70.4, SD = 20.3), with no significant between-group differences (expectancy: p = .095; perceived expectancy: p = .342).

**eExploratory Analyses**

**eExploratory Analyses on Social Exclusion**

The random intercept model of the outcome distress showed no Group × Time interaction effect, F(2, 131.56) = 0.36, p > .05, but a significant main effect of measurement time (F(2, 131.56) = 5.1, p = .026) with a marginal R-squared value of .02.

Exploratory analyses on the effect of the three recruitment waves on positive affect and distress showed no main effects. However, a significant main effect of recruitment wave on negative affect was found (*F*(2, 131.40) = 3.72, *p* = .027). Post-hoc pairwise contrasts revealed that the difference between recruitment wave one and recruitment wave three drove the main effect (*estimate* = 1.27, *SE* = 0.48, CI [0.32, 2.23], *t*[131.6] = 2.64, *p* = .009). As the model parameter showed, participants in the third recruitment wave reported significantly less negative affect in reaction to social exclusion than participants in the first recruitment wave (ß = −1.27, *SE* = 0.48, 95 % CI [−2.23, −0.32], *t*[131.62] = −2.64, *p* = .009). The model with the covariate recruitment wave explained more variance than the simpler models (*R²_m_* = .05). Exploratory analyses on sex differences revealed no effect of sex on positive or negative affect. For the outcome distress a significant main effect of sex was found (*F*(1, 131.73) = 4.19, *p* = .04). The model parameter indicated that women reported more distress than men (ß = 0.77, *SE* = 0.38, 95 % CI [0.03, 1.52], *t*[132.07] = 2.05, *p* = .043). The model with the covariate sex explained more variance than the simpler models (*R²_m_* = .04). Exploratory analyses on age differences revealed no significant effect of age on any of the subscales. Years of education did not predict positive affect and negative affect. However, a significant effect of years of education on distress was found (*F*(1, 131.5) = 5.68, *p* = .019). As the model parameter showed, more years of education were significantly associated with less distress (ß = −0.15, *SE* = 0.06, 95 % CI [−0.27, −0.03], *t*[131.5] = −2.38, *p* = .019). The model with the covariate years of education explained more variance than the simpler models (*R²_m_* = .05). Exploratory Pearson’s product-moment correlations between practice time and the outcomes showed no significant associations, neither for the meditation group (positive affect: *r*(42) = −.07, *p* = .64; negative affect: *r*(42) = .08, *p* = .60; distress: *r*(42) = −.006, *p* = .97), nor for the non-native language group (positive affect: *r*(42) = −.23, *p* = .13; negative affect: *r*(42) = .14, *p* = .35; distress: *r*(42) = .05, *p* = .75).

**eExploratory Analyses on Loneliness**

Exploratory analyses showed that neither recruitment wave, sex, age, nor years of education significantly predicted loneliness. Pearson’s product-moment correlation between practice time and loneliness of the intervention groups showed no associations (meditation group: r(43) = −.20, p = .181; non-native language group: r(43) = −.03, p = .826).
