# Supplementary material for: Meditation training and non-native language training both reduce older adults loneliness in the age-well randomized controlled trial
Source: Sci Rep. 2025 Sep 29;15:33332. doi: 10.1038/s41598-025-21058-7 (PMC12480588; doi:10.1038/s41598-025-21058-7)
Supplement: Supplementary file 2 — Supplementary Information 2. [file 41598_2025_21058_MOESM2_ESM.docx]

Supplemental Online Content: Nonauthor Collaborators

| **Group Name(s): Medit-Ageing Research Group** | | | | | | | |
| --- | --- | --- | --- | --- | --- | --- | --- |
| **First Name and Middle Initial(s)** | **Last Name** | **Suffix (eg, Jr, III)** | Academic Degrees | Institution | Location (city,  state/province, country) | Role or Contribution, eg, chair, principal investigator | Group (if more than 1  Group listed in the  byline) and/or Subgroup  (eg, Steering Committee) |
| Claire | André |  | PhD | INSERM | Caen, France | PhD student | NA |
| Martine | Batchelor |  |  | independent | Bordeaux, France | meditation teacher | NA |
| Axel | Beaugonin |  |  | independent | Caen/Paris, France | meditation teacher | NA |
| Pierre | Champetier |  | MSc | UNICAEN | Caen, France | PhD student | NA |
| Léa | Chauveau |  | MSc | INSERM | Caen, France | PhD student | NA |
| Anne | Chocat |  | MD | INSERM | Caen, France | investigating medical doctor | NA |
| Sophie | Dautricourt |  | MD PhD | UNICAEN | Caen/Lyon, France | PhD student | NA |
| Robin | De Flores |  | PhD | INSERM | Caen, France | post-doctoral position | NA |
| Vincent | De La Sayette |  | MD PhD | CHU Caen | Caen, France | Principal investigating medical doctor | NA |
| Pascal | Delamillieure |  | MD PhD | CHU Caen | Caen, France | meditation teacher | NA |
| Marion | Delarue |  | MSc | INSERM | Caen, France | neuropsychologist | NA |
| Hélène | Espérou |  | MD | INSERM | Caen, France | Study sponsor | NA |
| Séverine | Fauvel |  |  | INSERM | Caen, France | clinical research technician | NA |
| Francesca | Felisatti |  | MSc | INSERM | Caen, France | PhD student | NA |
| Eglantine | Ferrand-Devouge |  | MD | INSERM | Caen, France | investigating medical doctor | NA |
| Eric | Frison |  | MD PhD | INSERM | Bordeaux, France | methodologist | NA |
| Antoine | Garnier Crussard |  | MD MSc | CHU Lyon | Lyon, France | meditation teacher | NA |
| Francis | Gheysen |  | MD | independent | Caen, France | meditation teacher | NA |
| Sacha | Haudry |  | MSc | INSERM | Caen, France | PhD student | NA |
| Oriane | Hébert |  |  | INSERM | Caen, France | neuropsychologist | NA |
| Thien (Titi) | Huong Tran (Dolma) |  |  | INSERM | Paris, France | meditation teacher | NA |
| Elizabeth | Kuhn |  | PhD | INSERM | Caen, France | PhD student | NA |
| Brigitte | Landeau |  | MSc | INSERM | Caen, France | neuroimaging development engineer | NA |
| Florence | Mezenge |  | BA | INSERM | Caen, France | neuroimaging engineer assistant | NA |
| Cassandre | Palix |  | MSc | INSERM | Caen, France | PhD student | NA |
| Géraldine | Poisnel |  | PhD | INSERM | Caen, France | WP7 leader, research engineer | NA |
| Anne | Quillard |  | MD | INSERM | Caen, France | investigating medical doctor | NA |
| Géraldine | Rauchs |  | PhD | INSERM | Caen, France | researcher | NA |
| Corinne | Schimmer |  | MSc | UNICAEN | Caen, France | English teacher | NA |
| Edelweiss | Touron |  | MSc | INSERM | Caen, France | PhD student | NA |
| Anne-Laure | Turpin |  | MSc | INSERM | Caen, France | PhD student | NA |
| Caitlin | Ware |  | MSc | INSERM | Caen, France | English teacher | NA |
| Miranka | Wirth |  | PhD | DZNE | Dresden, Germany | WP2 deputy, researcher | NA |
